# Exploring the Influence of Mindful Self-Care on Workplace Engagement Among Nurses: A Path Analysis

**DOI:** 10.1097/jnr.0000000000000688

**Published:** 2025-06-25

**Authors:** Nasra ABDELHADI, Irit. BLUVSTEIN, Ronit KIGLI-SHEMESH, Semyon. MELNIKOV

**Affiliations:** 1 Department of Nursing Sciences, Steyer School of Health Professions, Gray Faculty of Medical and Health Sciences, Tel Aviv University, Tel Aviv, Israel; 2 The Herczeg Institute on Aging, Tel Aviv University, Tel Aviv, Israel; 3 Merhavim-Mental Health Center, Be’er Ya’akov Nes Ziona, Israel

**Keywords:** mindful self-care, compassion fatigue, nurses’ resilience, path analysis, work engagement

## Abstract

**Background:**

Work engagement in nurses is influenced by a variety of factors, with compassion fatigue identified as a negative predictor and resilience identified as a positive predictor. Although mindful self-care (MSC) may influence work engagement, this potential relationship has not been validated in the literature.

**Purpose:**

This study was designed to examine the relationship between MSC and work engagement in a sample population of nurses in Israel and to investigate the potential mediating effects of compassion fatigue, resilience, and internal health locus of control (IHLC) on this relationship.

**Methods:**

A quantitative cross-sectional study design was used, and data were collected from 845 nurses working in different clinical settings in Israel. A self-administered questionnaire was administered online between March and June 2023 to assess MSC, compassion fatigue, resilience, IHLC, and work engagement. A hypothesized model based on the Job Demands–Resources Theory was proposed. Descriptive statistics and path analysis were utilized in data analysis.

**Results:**

The proposed model demonstrated a good fit with the empirical data, explaining 17.2% of the variance in work engagement. Direct relationships were identified between work engagement and, respectively, MSC, compassion fatigue, resilience, and IHLC (β=−0.131, *p* < .01; β=0.011, *p* < .01; β=0.116, *p* < .05; β=0.280, *p* < .01, respectively). The relationship between MSC and work engagement was shown to be mediated by compassion fatigue and resilience (β=0.068, *p* < .01), while compassion fatigue was shown to partially mediate the relationship between MSC and resilience (β=0.025, *p* <01). IHLC was not found to be significantly associated with work engagement.

**Conclusions:**

MSC is a significant predictor of work engagement among nurses. Practicing MSC increases personal resilience and prevents compassion fatigue, leading to higher work engagement. Thus, we recommend nurse managers promote the regular practice of MSC, along with traditional self-care behaviors, among nurses to improve work engagement.

## Introduction

Work engagement is a persistent condition of vitality marked by substantial energy and psychological endurance at work as well as readiness to exert effort in one’s job. Work engagement encompasses commitment, distinguished by feelings of importance, excitement, motivation, pride, and a sense of being challenged. It also entails being fully immersed and intensely focused on one’s work tasks (Decuypere & Schaufeli, 2020). A recent study reported that nurses displaying greater levels of work engagement tended to report increased job satisfaction, improved quality of care, and a reduced inclination to quit (Wei et al., 2023).

Various elements have been recognized as factors contributing to work engagement in the nursing profession. Resilience and health locus of control (HLC) have been found to influence work engagement positively (Cabrera-Aguilar et al., 2023; Lui, 2019), while compassion fatigue has been found to be a factor that negatively impacts work engagement (Cao & Chen, 2021). During the COVID-19 pandemic, engaging in self-care practices such as maintaining online contact with coworkers and extended family, doing physical exercise at home, following a healthy diet, and engaging in activities that promote spiritual development (e.g., religion, meditation) have been shown to predict the vigor component of work engagement among health care professionals (Cuartero-Castañer et al., 2021). Another self-care practice, termed mindful self-care (MSC), is a recurring process that involves being mindfully aware of and evaluating one’s internal needs and external pressures, as well as deliberately engaging in self-care practices to meet and address those needs and pressures (Cook-Cottone & Guyker, 2017). However, no studies to date have examined the effect of MSC on work engagement among nurses or the interrelationships between work engagement and, respectively, MSC, compassion fatigue, HLC, and resilience.

### Mindful Self-Care

MSC has been found to improve professional quality of life and meaning among palliative care workers (Depner et al., 2021). Indeed, the implementation of MSC practices among inpatient nurses has led to higher job satisfaction, higher teamwork scores, and improved quality and safety metrics (Monroe et al., 2021). Moreover, Zeb et al. (2022) suggested that cultivating MSC gives nurses the opportunity to enhance their skills in building strong workplace relationships and elevate the care quality they provide to patients and their families. Therefore, MSC is hypothesized in this study as relating positively to the level of work engagement in nurses.

### Compassion Fatigue

Compassion fatigue, initially defined by Johnson (1992), refers to emotional, physical, and psychological weariness in health care providers associated with prolonged exposure to work stress (Johnson, 1992). Scholarly texts often blur the lines between compassion fatigue and secondary traumatic stress, with some considering secondary traumatic stress equivalent to compassion fatigue (Figley, 2013) and others viewing it either as a component of compassion fatigue (Stamm, 2010) or a broader category that also includes compassion fatigue (Brown et al., 2017). Thus, compassion fatigue and secondary traumatic stress are sometimes used synonymously (Hinderer et al., 2014). In this study, the term compassion fatigue is used. Mindful self-care rooted in self-awareness has been shown to ameliorate compassion fatigue (Delaney, 2018), which has been shown to relate negatively to both work engagement (Cao & Chen, 2021) and resilience (Zhang et al., 2022). However, no studies in the literature have assessed the relationships among MSC, compassion fatigue, resilience, and work fatigue. In this study, MSC in nurses is hypothesized as relating negatively to compassion fatigue, which in turn relates negatively to nurse resilience and work engagement.

### Personal Resilience

Resilience is defined as “stability in psychological adjustment following adversity or that resilience is common can carry negative consequences” (Infurna, 2020). Resilience emerges in professionals when they mentally process their experiences, frame their thoughts and emotions as a well-structured narrative, and develop a sense of meaning or purpose (Powell et al., 2020). Resilience has been shown to relate negatively to compassion fatigue and positively to work engagement (Cao & Chen, 2021). Therefore, in this study, MSC in nurses is hypothesized as being positively related to resilience, and resilience as being positively related to work engagement.

### Internal Health Locus of Control

The three dimensions of health locus of control include: (1) the internal health locus of control (IHLC) dimension, which involves the belief that health outcomes are influenced by an individual’s own abilities and efforts; (2) the powerful others dimension, which considers health outcomes to be determined by influential figures (e.g., physicians); and (3) the chance dimension, which attributes health outcomes to luck or fate (Wallston, 2005). Although a positive relationship among total health locus of control score, work motivation, and organizational commitment has previously been identified in nurses (Kalil et al., 2019), no study has examined the relationship between IHLC and work engagement. In this study, MSC is hypothesized to promote work engagement in nurses via IHLC.

The proposed relationships in this study among MSC, compassion fatigue, resilience, IHLC, and work engagement are based on Job Demands–Resources (JD-R) Theory (Bakker & Demerouti, 2017), which embodies the two key components of job demands and job resources. Job demands represent job elements such as high work pressure and challenging conditions that require continuous physical or mental effort and lead to physical/psychological strain. The other component, job resources, represents job elements such as autonomy, colleague support, and professional growth opportunities that facilitate goal attainment, reduce job demands, and promote growth and development. In this study, based on the JD-R Theory, personal resources such as MSC, resilience, and IHLC are presumed to buffer the impact of job demands (which may exacerbate compassion fatigue) to enhance work engagement.

The first aim of this study was to confirm whether MSC, resilience, and IHLC relate positively to work engagement and whether compassion fatigue relates negatively to work engagement. The second aim was to examine whether resilience, IHLC, and compassion fatigue mediate the MSC–work engagement relationship. The findings are expected to elucidate how these factors interact and influence each other in the context of nursing.

## Methods

### Design

A cross-sectional research design was used, and Strengthening the Reporting of Observational Studies in Epidemiology guidelines (von Elm et al., 2014) were followed. An online questionnaire was administered to nurses using Qualtrics software for data sampling and collection.

### Study Setting and Sampling

A convenience sample of nurses from different geographical regions of Israel was recruited, with 845 nurses enrolled as participants. The minimum number of participants required was determined as 129 using G*Power software, based on a linear multiple regression with four predictors, a medium effect size (*f* ^2^) of 0.15, a power of 0.95, and an α error of .05.

### Inclusion Criteria

The inclusion criteria were current employment as a nurse (regardless of age, gender, and job scope) and providing informed consent to participate.

### Study Instruments

The instruments originally in English with no Hebrew version were translated into Hebrew by the authors using the translation-back translation method.

#### Sociodemographics

Sociodemographic data collected included gender, age, marital/stable partner status, education (registered nurse, RN with a BA/BsN, MA, or PhD), and workplace (community, hospital, department, teaching, management, other).

#### Resilience

Resilience was assessed using the 10-item Connor–Davidson Resilience Scale (CD-RISC; Campbell-Sills & Stein, 2007), with items scored on a scale ranging from 0 (*not true at all*) to 4 (*true nearly all of the time*) and higher total scale scores indicating higher personal resilience. The Cronbach’s α value for the original CD-RISC was .85 (Campbell-Sills & Stein, 2007) and was .85 in this study as well.

#### Work engagement

Work engagement was assessed using the Utrecht Work Engagement Scale (UWES). This scale presumes that three dimensions, namely the physical (vigor), emotional (dedication), and cognitive (absorption), are the foundation of work engagement (Schaufeli et al., 2002). Vigor involves high energy, mental resilience, effort, and persistence in facing work challenges; dedication relates to deep involvement, feeling of significance, enthusiasm, and pride in work; and absorption involves being fully focused and engrossed in work, losing track of time, and finding it hard to detach from work tasks. The 9-item version of the Scale (UWES-9) was used in this study. The UWES-9 presents statements scored on a 7-point scale ranging from 0 (*never*) to 6 (*all of the time*). The Cronbach’s α for the UWES-9 was found to range from .89 to .97 (Schaufeli et al., 2002). Mean scores are used to calculate the final score, with higher scores indicating higher work engagement. In this study, the Cronbach’s α for the total scale was .86 and was .79, .84, and .65, respectively, for the vigor, dedication, and absorption subscales.

#### Internal health locus of control

IHLC was assessed using the 6-item Health Locus of Control Scale (Wallston, 2005), with item responses scored by level of agreement on a 6-point scale ranging from 1 (*strongly disagree*) to 6 (*strongly agree*). The cumulative score for each domain was used to calculate the total scale score, with higher scores indicating a stronger inclination toward IHLC. The Cronbach’s α coefficient for the HLC scale was found to range between .60 and .75 in prior research (Wallston, 2005) and was .74 in this study.

#### Compassion fatigue

Compassion fatigue was assessed using the secondary traumatic stress scale (Stamm, 2010). Scale items are scored on a 5-point Likert scale based on how accurately a statement reflects the respondent’s experience (i.e., 1 “*never*” to 5 “*very often*”). Item scores are summed to gain the total scale score, with ≤22 indicating a low level, 23–41 indicating an average level, and ≥42 indicating a high level of compassion fatigue (Stamm, 2010). The general reliability of the secondary traumatic stress scale was previously reported as .81 (Stamm, 2010) and was calculated as .87 in this study.

#### Mindful self-care

Cook-Cottone and Guyker (2017) developed the Mindful Self-Care Scale (MSCS) with the six subscales of physical care (eight items), supportive relationships (five items), mindful awareness (four items), self-compassion and purpose (six items), mindful relaxation (six items), and supportive structure (four items). Items are scored using a 5-point Likert scale that reflect the frequency (i.e., how much or how often) of a certain behavior during the past 1-week period, with 1=*never* (0 d), 2=*rarely* (1 d), 3=*sometimes* (2–3 d), 4=*often* (4–5 d), and 5=*regularly* (6–7 d). One item in the physical care subscale was reverse-scored. Subscale scores are calculated by averaging all items in each subscale, with mean subscale scores subsequently summed to calculate the total MSC score, with a total possible score range between 6 and 30. In the original study, Cronbach’s α scores for the subscales ranged between .68 and .94 (Cook-Cottone & Guyker, 2017) and were .84–.90 in this study.

### Data Collection

Data were collected between March and June 2023 after receiving approval from the Ethics Committee at Tel Aviv University. Master’s students in nursing shared the online questionnaire link and invitation to participate with their nurse friends via email or mobile phones. The study's purpose and instructions for filling out the questionnaire were described on the first page of the questionnaire. Potential participants were also informed that participation was completely voluntary, all questionnaire responses would remain anonymous and deidentified, and that all information provided would be used for research purposes only. They were also informed that, while they were free to stop completing the questionnaire at any point, all questions had to be answered to be included in the study.

### Data Analysis

SPSS Statistics Version 28 (IBM Corp., Armonk, NY, USA) was used to analyze the data. After assessing internal reliability using Cronbach’s α, a descriptive analysis was conducted, followed by independent *t* tests to examine sociodemographic variable–based differences in study variables (work engagement, resilience, IHLC, MSC, and compassion fatigue). Associations between variables were explored using Pearson correlations. *p* < .05 was set as the criterion for significance.

AMOS 28 was used to test the mediation hypotheses using structural equation modeling. Model fit to the data was tested using five goodness-of-fit indices. Two of these indices provided absolute measurements (i.e., the χ^2^ statistic and the Root Mean Square Error of Approximation [RMSEA]), while the Tucker-Lewis Index (TLI), Comparative Fit Index (CFI), and Normed Fit Index (NFI) were applied as incremental measures. Adequate model fit was indicated by RMSEA values <.08 and NFI, CFI, and TLI values >.90 (Preacher & Hayes, 2008). Next, a mediation model was used to examine the effects of compassion fatigue, resilience, and IHLC on the relationship between MSC and work engagement. Following Preacher and Hayes (2008), bootstrapping with 95% bias-corrected confidence intervals was employed to test both the direct and indirect effects of the mediation hypothesis. The bootstrapping process involved resampling the sample 5,000 times, allowing all direct and indirect estimates to be calculated. The *p* values for path coefficients and indirect effects were determined using 2-tailed tests in the bootstrapping process. Multiple indices were also examined to assess the model fit. Bootstrapping, a statistical resampling method, was instrumental in evaluating the mediation hypothesis.

### Ethical Considerations

Ethics approval to carry out this study was granted by Tel Aviv University’s ethics committee (approval number 0006284-2). Clicking the “I agree” button at the beginning of the electronic questionnaire was interpreted as providing informed consent and was necessary to begin filling out the questionnaire.

## Results

Eight hundred and forty-five nurses were enrolled as participants. The distributions of participants across different sociodemographic variables are summarized in Table 1. Most (80%) of the participants were female, the mean age was 37.1 (*SD*=10.61; 21.0–72.0) years, a majority were Jews (72%) and a minority were Arabs (23.7%), most (77.8%) reported having a stable partner, and most held a BA/BScN degree (58.9%) and worked in hospitals (72.4%).

**Table 1:** Sociodemographic Variables (N=845)

Variable	*n* (%)
Gender	
Male	162 (19.2)
Female	679 (80.4)
Other	2 (0.2)
Missing	2 (0.2)
Marital status	
Stable partner	657 (77.8)
No stable partner	183 (21.7)
Missing	5 (0.6)
Age (year; *M* and *SD*)	37.1 (10.61)
Median (range)	35.0 (21.0–72.0)
Seniority (year; *M* and *SD*)	11.35 (10.7)
Median (range)	7.0 (0–45.0)
Ethnicity	
Jew	608 (72.0)
Arab	200 (23.7)
Other	27 (3.2)
Missing	10 (1.2)
Educational level	
RN	88 (10.4)
RN BA/BSN	498 (58.9)
RN MA/PhD	249 (29.5)
Workplace setting	
Community clinic	141 (16.7)
Hospital	612 (72.4)
Management	14 (1.7)
Other	77 (9.1)
Missing	1 (0.1)

*Note.* BA = Bachelor of Arts; BSN = Bachelor of Science in Nursing; MA = Master of Arts; PhD = Doctor of Philosophy; RN = Registered Nurse.

Questionnaire scores are summarized in Table 2. The mean scores for total work engagement and MSC were 4.57 (*SD*=0.96) and 20.19 (*SD*=3.24), respectively. Correlations among the study variables are presented in Table 3. A statistically significant, positive correlation was identified between work engagement and, respectively, resilience (*r*_p_=.306, *p* < .01), IHLC (*r*_p_=.086, *p* < .05), compassion fatigue (*r*_p_=−.105, *p* < .01) and MSC (*r*_p_=.344, *p* < .01).

**Table 2:** Participant Questionnaire Scores (*N* = 845)

Variable	*M* (*SD*)	Observed Range	Median
Work engagement	4.57 (0.96)	0.75–6.00	4.78
Vigor	4.54 (1.12)	0.00–6.00	5.00
Dedication	4.71 (1.19)	0.00–6.00	5.00
Absorption	4.46 (1.12)	0.00–6.00	4.67
Resilience	5.90 (1.40)	0.00–8.00	6.00
Internal health locus of control	24.31 (4.87)	6.00–36.00	25.00
Compassion fatigue	21.22 (5.61)	9.00–39.00	21.00
Mindful self-care (total)	20.19 (3.24)	6.50–29.50	20.29
Physical care	2.78 (0.80)	1.13–5.00	2.75
Supportive relationships	3.79 (0.73)	1.00–5.00	3.80
Mindful awareness	3.46 (0.78)	1.00–5.00	3.50
Self-compassion and purpose	3.58 (0.70)	1.00–5.00	3.67
Mindful relaxation	3.06 (0.75)	1.00–5.00	3.00
Supportive structure	3.62 (0.75)	1.00–5.00	3.75

**Table 3:** Correlations Among Questionnaire Variables (N=845)

Variable	1	2	3	4
1. Work engagement	—			
2. Resilience	.306**	—		
3. Internal health locus of control	.086*	.034	—	
4. Compassion fatigue	−.105**	−.226**	.020	—
5. Mindful self-care	.344**	−.258**	.118**	−.128**

* *p*<.05. **p<.01.

**Figure 1:**
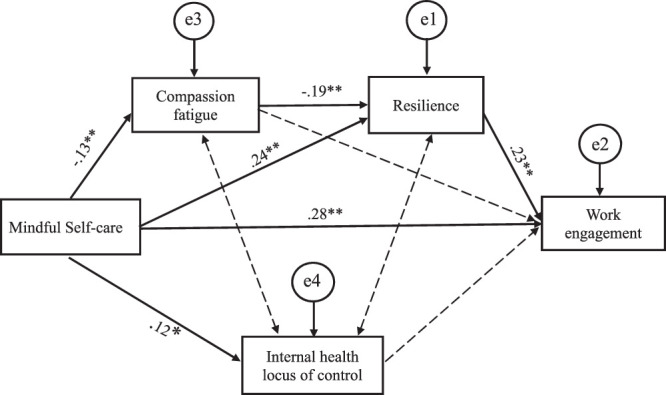
Relationships Among MSC, Compassion Fatigue, Resilience, Internal Health Locus of Control, and Work Engagement: A Path Analysis

*Note.* Standardized estimates, Default model, *χ* ^2^ = 1.174, χ^2^/df = 0.587, *p* = .556, degrees of freedom = 2, RMSEA < .001, dashed lines show non-significant correlation. *p < .05. **p < .01.

In terms of assessing the mediating roles of compassion fatigue, resilience, and IHLC on the relationship between MSC and work engagement, the fit measures for the path model indicate a good fit (χ^2^=1.174, χ^*2*^ */df*=0.587, *p*=.556, degrees of freedom=2, NFI=.996, CFI=1.000, and TLI=1.015, RMSEA<.001 and SRMR=.0091). The model accounted for 17.2% of the variance in work engagement. The results of the path analysis indicate a significantly positive, direct effect of MSC on work engagement (β=.280, *p* = .006) and negative direct effects of MSC on compassion fatigue (β=−.131, *p* = .006), and compassion fatigue on resilience (β=−.195, *p* = .015). Also, a significantly positive, direct effect of MSC on resilience (β=.239, *p* = .008), and IHLC (β=.116, *p* = .016) was found (Table 4). Resilience was shown to positively and directly influence work engagement (β=.227, *p* = .009), while no direct effects of compassion fatigue on either work engagement or of IHLC on work engagement were found. Moreover, the path analysis results show a significantly positive, indirect effect of MSC on work engagement through the variables of compassion fatigue and resilience (β=.068, *p* =.005); a significantly positive, indirect effect of MSC on resilience through the variable of compassion fatigue (β=.025, *p* = .007); and a significantly negative correlation between compassion fatigue and work engagement through the variable of resilience (β=−.044, *p* = .013). IHLC was not found to mediate the relationship between MSC and work engagement. An AMOS diagram of the path analysis is presented in Figure 1.

**Table 4:** Direct and Indirect Effects Among the Research Variables

Standardized Direct and Indirect Effects	Bootstrapping		Bias-Corrected 95% CI		*p*
	β	*SE*	Lower	Upper	
Direct effect					
MSC–Compassion fatigue	−.131	0.006	−0.198	−0.058	**.006**
Compassion fatigue–Resilience	−.195	0.007	−0.256	−0.127	**.015**
Compassion fatigue–IHLC	.020	0.101	−0.003	0.011	.322
IHLC–Resilience	.011	0.010	−0.013	0.020	.706
MSC–Resilience	.239	0.014	0.176	0.319	**.008**
MSC–IHLC	.116	0.052	0.054	0.176	**.016**
MSC–Work Engagement	.280	0.010	0.218	0.345	**.006**
Resilience–Work Engagement	.227	0.023	0.176	0.289	**.009**
IHLC–Work Engagement	.047	0.006	−0.002	0.125	.119
Compassion fatigue–Work engagement	−.018	0.053	−0.069	0.030	.531
Indirect effect					
MSC–Compassion fatigue–Resilience	.025	—	0.010	0.044	**.007**
MSC–Compassion fatigue–Work Engagement	.002	—	−0.004	0.011	.516
Compassion fatigue–Resilience–Work Engagement	−.044	—	−0.060	−0.027	**.013**
MSC–Compassion fatigue–Resilience–Work engagement	.068	—	0.050	0.092	**.005**
MSC–IHLC–Work Engagement	.005	—	−0.001	0.014	.226

*Note. R* ^2^ for Work engagement = .172. Bold values indicate statistically significant results (*p* < .05). IHLC = internal health locus of control; MSC = mindful self-care.

## Discussion

This study was designed to investigate the relationship between MSC and workplace engagement in a national sample of nurses, as well as to determine whether compassion fatigue, resilience, and/or IHLC mediated this relationship. The results of the data analysis indicate that MSC and workplace engagement are significantly related, and that this relationship is partially mediated by compassion fatigue and resilience. Therefore, the findings support direct and indirect (mediated) relationships between MSC and work engagement in nurses.

In this study, the mean total work engagement score was 4.57 (*SD*=0.96), with mean scores for the subscales of vigor, dedication, and absorption ranging between 4.46 and 4.71. These scores are higher than those found in a pre–COVID-19 pandemic sample of nurses (Navarro-Abal et al., 2023) and similar to those in a sample of nurses studied during the COVID-19 pandemic (Allande-Cussó et al., 2021). Because this study was conducted after the COVID-19 pandemic, one possible explanation for the high work engagement scores may be that the pandemic experience promoted self-reflection and growth, which impacted work engagement levels. The higher scores in this study compared to those surveyed during the pre-pandemic period may also reflect the effect of nurse adaptation and resilience mechanisms. Moreover, the total (*SD*) level of MSC in this study was 20.19 (*SD*=3.24) with the scores for the six MSC subscales ranging between 2.78 and 3.79. These scores are lower than those found in a previous study among nurses conducted before the COVID-19 pandemic (Vannucci & Weinstein, 2017) and higher than those reported by health care professionals during the COVID-19 pandemic (Osman & Singaram, 2023). The differences in total MSC score and scores for the six MSC subscales may be attributable to the impact of the pandemic on mental health and well-being worldwide due to direct pandemic impacts, the different stress and coping mechanisms used by health care professionals, and cultural differences in how MSC is perceived, valued, and practiced.

The direct association found in this study between MSC and work engagement has never been previously reported, although prior studies have examined associations between MSC and variables related to work engagement. Depner et al. (2021) found that greater commitment to MSC predicted greater meaning and a higher professional quality of life among palliative care workers (Depner et al., 2021). In addition, low MSC scores among nurses were shown to have a potentially negative effect on their care and interactions with patients and patient families (Zeb et al., 2022). Nurses working in an inpatient unit where MSC practices were implemented experienced notably higher levels of job satisfaction, and while not statistically significant, also ranked higher in teamwork compared to nurses in units that did not implement MSC practices (Monroe et al., 2021). The results of this study show a direct link between MSC and work engagement, which, coupled with prior findings that connect MSC to aspects influencing work engagement, such as professional quality of life, interactions with patients and their families, and job satisfaction, underscore the importance of MSC practices among nurses. These practices not only enhance the work engagement and capacity of nurses but also positively impact their patient care quality and job satisfaction (Monroe et al., 2021).

Furthermore, the results support that compassion fatigue mediates the relationship between MSC and resilience. Notably, compassion fatigue and personal resilience also mediated the relationship between MSC and work engagement. These are novel findings. According to the Compassion Fatigue Resilience Model (Schwanz & Paiva‑Salisbury, 2022), the self-care behaviors and self-care beliefs of health care professionals relate negatively to compassion fatigue and positively with compassion satisfaction, which, in turn, promote resilience against compassion fatigue (Schwanz & Paiva‑Salisbury, 2022). In this study, compassion fatigue was found to mediate the relationship between MSC and resilience. It seems that through MSC, the participants experienced lower compassion fatigue and higher compassion satisfaction, which in turn led to higher resilience. The results also show that resilience mediated the relationship between compassion fatigue and work engagement, which is similar to the finding reported by Labrague and de Los Santos (2021) that resilience mediated the relationship between compassion fatigue and job outcomes (e.g., job satisfaction, intention to leave, quality of care provided during the pandemic) in frontline nurses (Labrague & de Los Santos, 2021). Also similar to this study, a study of palliative care providers during the COVID-19 pandemic found positive associations among MSC, resilience, and professional life satisfaction. In that same study, those subjects with greater resilience had longer tenures in palliative care (Garcia et al., 2022). Lastly, an MSC and resiliency intervention was shown to promote significant improvements in burnout and stress scores among nurses (Craigie et al., 2016). These results may be explained by the JD-R Theory when personal resources (e.g., MSC, resilience, IHLC) act as a buffer against the effects of job demands that may promote compassion fatigue and thus work engagement. In conclusion, the work done in this study identified a new relationship pathway linking MSC, compassion fatigue, resilience, and work engagement.

Notably, no correlation was found between IHLC and work engagement. This contradicts Kalil et al. (2019), who found significant positive relationships among locus of control, motivational factors, and organizational commitment in nurses (Kalil et al., 2019). This difference may be explained by the different study populations used. For instance, their specific characteristics and experiences may have made the nurse subjects in Kalil et al. more responsive to locus of control-related factors than the participants in this study. Also, the different assessment tools used may capture different aspects of these constructs. Future studies should include a broader range of participants from various health care settings, cultures, and backgrounds to better understand the relationship between IHLC and work engagement.

### Strengths and Limitations

Despite the long-recognized importance of MSC, this study was the first to examine the relationship between MSC and workplace engagement. Moreover, the study design and use of path analysis to elucidate how variables interact by analyzing their direct dependency should be viewed as strengths. However, several limitations should be noted. Employing a cross-sectional research approach restricts the potential to deduce causal connections. Also, using a convenience sample of nurses from various regions in Israel may limit the generalizability of the findings. Relying on self-report questionnaires increases the risk of social desirability, response, and other biases. Furthermore, the study’s findings are specific to the Israeli health care context and may not be generalizable to other cultural or geographical settings.

Longitudinal studies would be more effective in determining the directionality and causality of the relationships between variables. Further, future studies may benefit from using random or stratified sampling methods to enhance representativeness and increase generalizability. In addition, future studies may either incorporate more objective measures or triangulate data using qualitative interviews and observations.

### Implications for Policy and Practice

The findings reinforce the widespread presumption that nurses enhance the effectiveness of care, improve the quality of care, and enhance work engagement in health care organizations. Engaging in MSC and strengthening resilience can positively influence engagement at work. Accordingly, supporting MSC and addressing key challenges are essential tasks that nursing policymakers should undertake to alleviate compassion fatigue and improve care quality. In addition, health care providers should consider that mindfulness is often associated with a high level of personal resilience, which can also further enhance work engagement in nurses.

### Conclusions

The level of personal resilience in hospital nurses and the level of compassion fatigue they experience while treating patients were confirmed in this study to significantly influence the relationship between mindful self-care and work engagement. Based on these findings, personal resilience in the context of mindful self-care represents an important influence on the work engagement level of clinical nurses. As demonstrated by several goodness-of-fit indices, the mediation analysis conducted using structural equation modeling indicates a good fit between the model and the data. Accordingly, the proposed model adequately explains the relationships among the study variables.

### Author Contributions

Study conception and design: IB, RKS, SM

Data collection: IB, RKS, SM

Data analysis and interpretation: All authors

Drafting of the article: NA

Critical revision of the article: SM

## References

[R1] Allande-Cussó R. García-Iglesias J. J. Ruiz-Frutos C. Domínguez-Salas S. Rodríguez-Domínguez C. Gómez-Salgado J. (2021). Work engagement in nurses during the Covid-19 pandemic: A cross-sectional study. Healthcare, 9(3), Article 253. https://doi.org/10.3390/healthcare9030253 33804351 10.3390/healthcare9030253 PMC8001401

[R2] Bakker A. B. Demerouti E. (2017). Job demands-resources theory: Taking stock and looking forward. Journal of Occupational Health Psychology, 22(3), 273–285. https://doi.org/10.1037/ocp0000056 27732008 10.1037/ocp0000056

[R3] Brown J. L. C. Ong J. Mathers J. M. Decker J. T. (2017). Compassion fatigue and mindfulness: Comparing mental health professionals and MSW student interns. Journal of Evidence-Informed Social Work, 14(3), 119–130. https://doi.org/10.1080/23761407.2017.1302859 28388339 10.1080/23761407.2017.1302859

[R4] Cabrera-Aguilar E. Zevallos-Francia M. Morales-García M. Ramírez-Coronel A. A. Morales-García S. B. Sairitupa-Sanchez L. Z. Morales-García W. C. (2023). Resilience and stress as predictors of work engagement: The mediating role of self-efficacy in nurses. Frontiers in Psychiatry, 14, Article 1202048. https://doi.org/10.3389/fpsyt.2023.1202048 37649562 10.3389/fpsyt.2023.1202048 PMC10464840

[R5] Campbell-Sills L. Stein M. B. (2007). Psychometric analysis and refinement of the Connor-Davidson Resilience Scale (CD-RISC): Validation of a 10-item measure of resilience. Journal of Traumatic Stress, 20(6), 1019–1028. https://doi.org/10.1002/jts.20271 18157881 10.1002/jts.20271

[R6] Cao X. Chen L. (2021). Relationships between resilience, empathy, compassion fatigue, work engagement and turnover intention in haemodialysis nurses: A cross-sectional study. Journal of Nursing Management, 29(5), 1054–1063. https://doi.org/10.1111/jonm.13243 33393134 10.1111/jonm.13243

[R7] Cook-Cottone C. P. Guyker W. M. (2017). The development and validation of the Mindful Self-Care Scale (MSCS): An assessment of practices that support positive embodiment. Mindfulness, 9(1), 161–175. 10.1007/s12671-017-0759-1

[R8] Craigie M. Slatyer S. Hegney D. Osseiran-Moisson R. Gentry E. Davis S. Dolan T. Rees C. (2016). A pilot evaluation of a Mindful Self-care and Resiliency (MSCR) intervention for nurses. Mindfulness, 7(3), 764–774. 10.1007/s12671-016-0516-x

[R9] Cuartero-Castañer M. E. Hidalgo-Andrade P. Cañas-Lerma A. J. (2021). Professional quality of life, engagement, and self-care in healthcare professionals in Ecuador during the COVID-19 pandemic. Healthcare, 9(5), Article 515. 10.3390/healthcare9050515 33946629 PMC8146458

[R10] Decuypere A. Schaufeli W. (2020). Leadership and work engagement: Exploring explanatory mechanisms. German Journal of Human Resource Management, 34(1), 69–95. 10.1177/2397002219892197 (Original work published in Deutsch)

[R11] Delaney M. C. (2018). Caring for the caregivers: Evaluation of the effect of an eight-week pilot mindful self-compassion (MSC) training program on nurses’ compassion fatigue and resilience. PLOS ONE, 13(11), Article e0207261. 10.1371/journal.pone.0207261 30462717 PMC6248952

[R12] Depner R. M. Cook-Cottone C. P. Kim S. (2021). Structural relationship between mindful self-care, meaning made, and palliative worker’s quality of life. International Journal of Stress Management, 28(1), 74–87. 10.1037/str0000209

[R13] Figley C. R. (2013). Compassion fatigue: Coping with secondary traumatic stress disorder in those who treat the traumatized. Routledge. 10.4324/9780203777381

[R14] Garcia A. C. M. Ferreira A. C. G. Silva L. S. R. da Conceição V. M. Nogueira D. A. Mills J. (2022). Mindful self-care, self-compassion, and resilience among palliative care providers during the COVID-19 pandemic. Journal of Pain and Symptom Management, 64(1), 49–57. 10.1016/j.jpainsymman.2022.03.003 35292366 PMC8917778

[R15] Hinderer K. A. VonRueden K. T. Friedmann E. McQuillan K. A. Gilmore R. Kramer B. Murray M. (2014). Burnout, compassion fatigue, compassion satisfaction, and secondary traumatic stress in trauma nurses. Journal of Trauma Nursing, 21(4), 160–169. 10.1097/JTN.0000000000000055 25023839

[R16] Infurna F. J. (2020). What does resilience signify? An evaluation of concepts and directions for future research. Gerontology, 66(4), 323–331. 10.1159/000507365 32408298

[R17] Johnson C. (1992). Coping with compassion fatigue. Nursing, 22(4), 116–121. 1570090

[R18] Kalil S. I. M. Abd-Elrhaman E. S. A. Sliman W. M. M. (2019). Relationship among nurses’ locus of control, work motivation factors, and their organizational commitment. American Journal of Nursing Research, 7(2), 167–178. 10.12691/ajnr-7-2-8

[R19] Labrague L. J. de Los Santos J. A. A. (2021). Resilience as a mediator between compassion fatigue, nurses’ work outcomes, and quality of care during the COVID-19 pandemic. ANR: Applied Nursing Research, 61, Article 151476. 10.1016/j.apnr.2021.151476 34544570 PMC8448586

[R20] Lui N. M. J. (2019). Organizational and individual work-related factors on longitudinal nurse employee outcomes and productivity costs [Master’s thesis]. The University of Hong Kong.

[R21] Monroe C. Loresto F. Horton-Deutsch S. Kleiner C. Eron K. Varney R. Grimm S. (2021). The value of intentional self-care practices: The effects of mindfulness on improving job satisfaction, teamwork, and workplace environments. Archives of Psychiatric Nursing, 35(2), 189–194. 10.1016/j.apnu.2020.10.003 33781399 PMC7553100

[R22] Navarro-Abal Y. Climent-Rodríguez J. A. Vaca-Acosta R. M. Fagundo-Rivera J. Gómez-Salgado J. García-Iglesias J. J. (2023). Workplace violence: Impact on the commitment and involvement of nurses at work. Journal of Nursing Management, 2023(1), Article 9987092. 10.1155/2023/9987092 40225620 PMC11918937

[R23] Osman I. Singaram V. S. (2023). Effects of personality traits on mindful self-care practices of healthcare workers. South African Journal of Psychiatry, 29, Article 2019. 10.4102/sajpsychiatry.v29i0.2019 PMC10091165 37064752

[R24] Powell M. J. Froggatt K. Giga S. (2020). Resilience in inpatient palliative care nursing: A qualitative systematic review. BMJ Supportive & Palliative Care, 10(1), 79–90. 10.1136/bmjspcare-2018-001693 30808628

[R25] Preacher K. J. Hayes A. F. (2008). Asymptotic and resampling strategies for assessing and comparing indirect effects in multiple mediator models. Behavior Research Methods, 40(3), 879–891. 10.3758/BRM.40.3.879 18697684

[R26] Schaufeli W. B. Salanova M. González-romá V. Bakker A. B. (2002). The measurement of engagement and burnout: A two sample confirmatory factor analytic approach. Journal of Happiness Studies, 3, 71–92. 10.1023/a:1015630930326

[R27] Schwanz K. A. Paiva-Salisbury M. (2022). Before they crash and burn (out): A compassion fatigue resilience model. Journal of Wellness, 3(3), Article 7. 10.55504/2578-9333.1100

[R28] Stamm B. (2010). The concise ProQOL manual (2nd ed.). ProQOL.org.

[R29] Vannucci M. J. Weinstein S. M. (2017). The nurse entrepreneur: Empowerment needs, challenges, and self-care practices. Nursing: Research and Reviews, 7, 57–66. 10.2147/NRR.S98407

[R30] von Elm E. Altman D. G. Egger M. Pocock S. J. Gøtzsche P. C. Vandenbroucke J. P. STROBE Initiative (2014). The Strengthening the Reporting of Observational Studies in Epidemiology (STROBE) statement: Guidelines for reporting observational studies. International Journal of Surgery, 12(12), 1495–1499. 10.1016/j.ijsu.2014.07.013 25046131

[R31] Wallston K. A. (2005). The validity of the multidimensional health locus of control scales. Journal of Health Psychology, 10(5), 623–631. 10.1177/1359105305055304 16033784

[R32] Wei H. Horsley L. Cao Y. Haddad L. M. Hall K. C. Robinson R. Powers M. Anderson D. G. (2023). The associations among nurse work engagement, job satisfaction, quality of care, and intent to leave: A national survey in the United States. International Journal of Nursing Sciences, 10(4), 476–484. 10.1016/j.ijnss.2023.09.010 38020845 PMC10667320

[R33] Zeb H. Arif I. Younas A. (2022). Mindful self-care practice of nurses in acute care: A multisite cross-sectional survey. Western Journal of Nursing Research, 44(6), 540–547. 10.1177/01939459211004591 33825565

[R34] Zhang J. Wang X. Xu T. Li J. Li H. Wu Y Li Y. Chen Y. Zhang J.-P. (2022). The effect of resilience and self-efficacy on nurses’ compassion fatigue: A cross-sectional study. Journal of Advanced Nursing, 78(7), 2030–2041. 10.1111/jan.15113 34825731

